# Ultra-fast and automated immunohistofluorescent multistaining using a microfluidic tissue processor

**DOI:** 10.1038/s41598-019-41119-y

**Published:** 2019-03-14

**Authors:** Giulia Cappi, Diego Gabriel Dupouy, Marta Aurelia Comino, Ata Tuna Ciftlik

**Affiliations:** Lunaphore Technologies SA, EPFL Innovation Park, Building C, 1015 Lausanne, Switzerland

## Abstract

Multistaining of a tissue section targeting multiple markers allows to reveal complex interplays in a tumor environment. However, the resource-intensive and impractically long nature of iterative multiplexed immunostainings prohibits its practical implementation in daily routine, even when using work-flow automation systems. Here, we report a fully automated and ultra-fast multistaining using a microfluidic tissue processor (MTP) in as short as 20 minutes per marker, by immunofluorescent staining employing commercially available tyramide signal amplification polymer precipitation by horse-radish peroxidase (HRP) activation. The reported duration includes (i) 15 minutes for the entire fluidic exchange and reagent incubation necessary for the immunostaining and (ii) 5 minutes for the heat-induced removal of the applied antibodies. Using the automated MTP, we demonstrated a 4-plex automated multistaining with clinically relevant biomarkers within 84 minutes, showing perfect agreement with the state-of-the-art microwave treatment antibody removal. The presented HRP-based method is in principle extendable to multistaining by both tyramides accommodating higher number of fluorescent channels and multi-color chromogenic staining. We anticipate that our automated multi-staining with a turn-around time shorter than existing monoplex immunohistochemistry methods has the potential to enable multistaining in routine without disturbing the current laboratory workflow, opening perspectives for implementation of -omics approaches in tissue diagnostics.

## Introduction

Nowadays the state-of-the art of immunohistochemistry (IHC) is being challenged more and more with the increasing need for precision in molecular subtyping of cancers. Recent trends in personalized medicine suggest that a higher number of biomarker tests allows a more precise diagnosis, and eventually higher treatment success^1^. Already, the detection of multiple markers for a single patient is often required for clinical purposes^2–5^ and it is common practice to use several adjacent tissue sections for each staining to complete a diagnosis. However, often the spatial morphology of the tissue evolves over the cuts thus not providing the same information across the whole set of adjacent tissue sections, while missing co-expression of markers in the same cells. Recently, multi-staining kits using the precipitation of 3-color chromogens are being increasingly used for research purposes, such as the DISCOVERY kit from Roche Ventana^6^. Similarly, in addition to the current diagnostic practice, immunophenotyping, which comprises monitoring the expression of several biomarkers related with tumor infiltrating lymphocytes (TILs) and their interaction with the tumor, is an emerging technique because of its potential impact in cancer immunotherapy research and potential diagnostic application^7,8^. Consequently, the availability of a tissue staining technique that would enable widespread and routine utilization of multiplexed immunohistostaining is becoming increasingly crucial for diagnostic and clinical research purposes.

To date, two major methods for multiplexed immunostaining have been introduced: spatial and iterative multiplexing. Spatial multiplexing, in principle, allows staining of spatially different locations of the tissue section to increase the number of biomarkers on a single section. Kim *et al*. presented a multiplexed approach called multiplexed microfluidic IHC platform^9^ that consists of 10 small (300 *μ*m) adjacent channels for searching different markers in spatially different locations. Similarly, IBM research presented a device called microfluidic probe, where vertical microfluidic holes are arranged inside a very small spot of about 100 *μ*m in diameter to stain regions of interest in a tissue or cell monolayer. Using the microfluidic probe top, spatial multiplexing for 4 different antibodies was demonstrated by moving the probe head^10^. Nevertheless, with the use of spatial multiplexing, staining a clinically relevant area of the tissue might be impractically long, and a partial staining area for each marker would not be clinically relevant. Hence, this technique is potentially not suitable for integration in a routine laboratory workflow.

Iterative multiplexing, on the other hand, can stain the entire tissue section and preserve the morphological context to produce clinically relevant results. Image-based iterative multiplexing has been shown to simultaneously detect up to 50 biomarkers, where each immunostaining cycle includes (i) a first immunostaining, (ii) an intermittent imaging of the tissue, (iii) removal or inactivation of the stainings^11–15^. Yet, the intermittent imaging requires either manual intervention for image acquisition in-between staining cycles, which results in long turnaround times (TAT), or integrated staining-imaging platforms that are expensive and not widespread. In order to overcome this, tyramide signal amplification (TSA) was introduced in multiplexing assays, where each immunostaining results in a precipitated fluorescent polymer over the tissue. Such TSA-precipitate remains on the slide during the removal of the antibodies, and multiple staining and antibody removal cycles result in the accumulation of different color TSA-precipitates. Using TSA-based multiplexing, up to 7 different colors are shown, leveraging advanced multispectral analysis^16^, automated or semi-automated protocols on state-of-the-art staining equipment^17^ (and final microscope check by the pathologist on the stained slides).

In this context, TSA-based multiplexing could be the most suitable method to replace standard IHC in the laboratory workflow when co-localized staining of multiple markers is required for diagnosis. Yet, the introduction of multiplexed stainings comprising a high number of markers per case requires high-duty utilization of laboratory resources and automated staining equipment. For example, compared to a monoplex IHC, a 7-plex staining would imply an order of magnitude higher TAT (the shortest reported to be 2.5 hours per marker^17^) and staining equipment occupation in an anatomical pathology laboratory, potentially impeding its widespread use and implementation^17,18^. What is more, such multiplexed stained tissue requires digital scanning of the slides before the observation by a pathologist. Therefore, if a multiplexed TSA-based staining could be rendered faster than the current monoplex IHC TAT of between 2.5 to 4 hours, it could seemingly be integrated to the current anatomical pathology workflow and may enable the routine use of multiplexed immunostaining.

**Figure 1:**
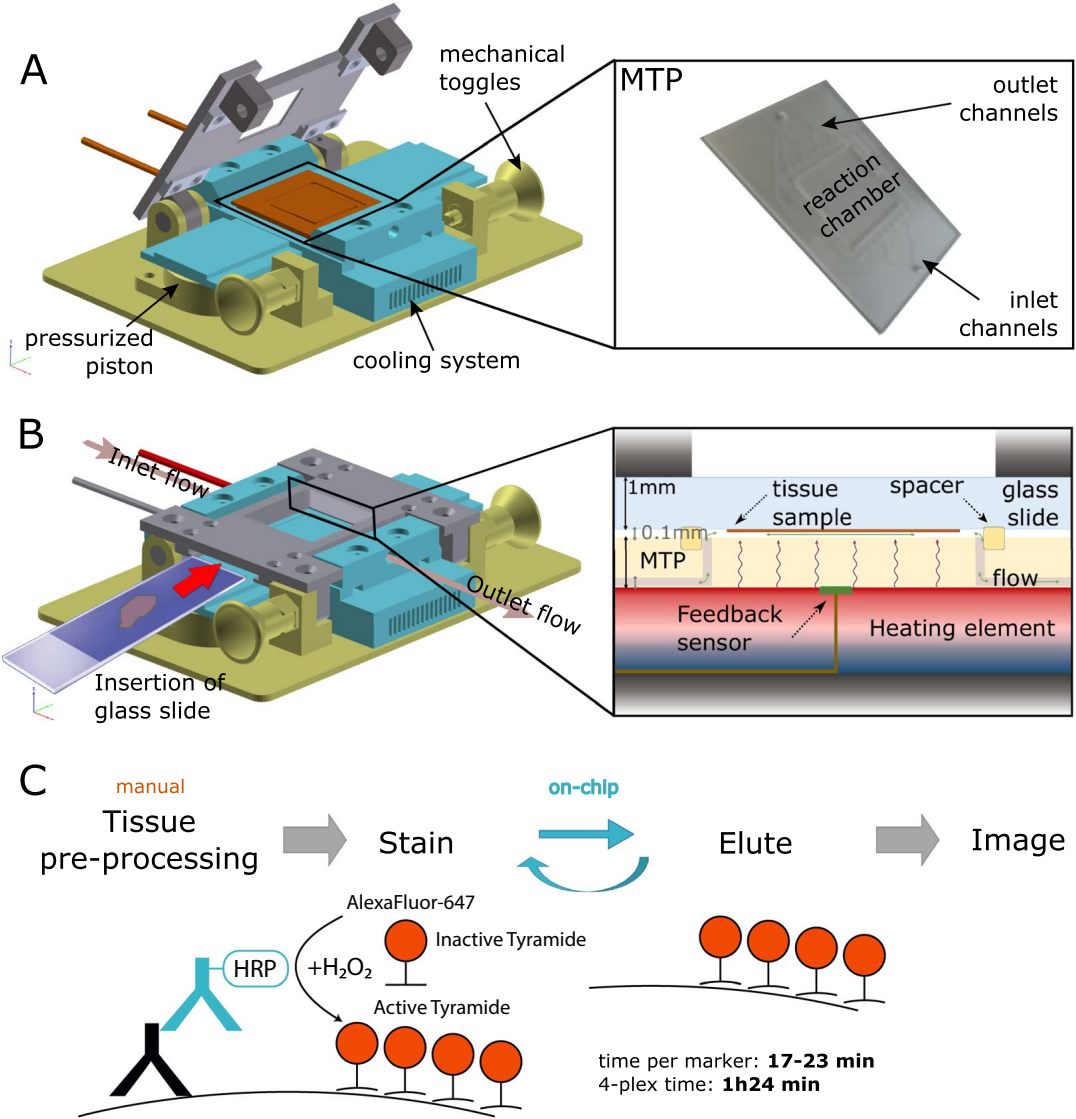
Microfluidic multiplexing working principle. (**A**) Sketch of the open stainer showing the position of the MTP inside it. Two mechanical toggles allow for easy opening and closing of the platform. Inset: picture of the MTP. The inset shows the details of the microfluidic inlet and outlet channels. (**B**) Schematics of the closed stainer and loading of the microscope slide. The slide is inserted laterally such that the tissue is positioned above the reaction chamber. Underneath, a pressurized piston exerts the necessary force to seal the chamber for fast fluid exchange. The inset shows a cut view of the reaction chamber, where the microscope slide hosting the tissue section is clamped together with the MTP chip. The MTP is supported by the heating element, electronically controlled to provide or remove heat from its surface. Cooling grooves facilitate heat dissipation. (**C**) Work flow of the staining protocol based on the TSA detection system. (i) Tissue pre-processing: slides are manually dehydrated, deparaffinized, re-hydrated and processed for heat-induced epitope retrieval (HIER); (ii) On-chip staining and antibody removal cycles take from 17 to 23 minutes per marker, reaching a total time of 1 h 24 min for a 4-plex staining. The steps performed on-chip are detailed in Table 1; (iii) Slides are finally removed from the stainer, coverslipped and scanned using a multi-spectral epifluorescence microscope. Mechanical stainer designed by Marco Ammann, Lunaphore Technologies SA.

Here, we report fully automated and ultra-fast TSA-based multi-staining in as short as 20 minutes average per marker (Fig. 1) using a microfluidic tissue processor (MTP) that we have recently introduced^19,20^. The technique requires only commercially available primary antibodies and TSA kits, and the reported duration includes (i) 15 minutes average for the entire fluidic exchange and reagent incubations necessary for TSA-based immunostaining and (ii) heat-induced antibody removal in 5 minutes, including heating and cooling cycles. We first introduce an ultra-fast antibody removal that takes the advantage of the fast heat exchange in the microfluidic reaction chamber, which reveals comparable results to the widely used microwave treatment (MWT) antibody removal^16,17,21^. Later, in a proof-of-concept experiment, we demonstrated a 4-plex automated multi-staining on a breast cancer TMA within 84 minutes including counterstaining. The presented method is in principle extendable up to 7-plex by using commercial spectral demultiplexing solutions^22^ with a TAT of around 2 hours. To our knowledge, this is the first time that a multistaining assay can be fully run in automated fashion in less than 20 minutes per marker, also leaving sufficient time for digital slide scanning after the staining. We anticipate that the reported technique opens perspectives for general use of multistaining in traditional IHC biomarker panels, the practical and widespread implementation of immunophenotyping, and other -omics like approaches in tissue immunostaining in the longer term.

## Results

### Fast heat exchange allows ultra-fast antibody heat-induced removal in 5 minutes

The fast fluid exchange technology is employed as a key element to ensure fast and uniform coloration of the tissue. As depicted in Fig. 1A,B, with the addition of a temperature control and a cooling system, a fast heat exchange is achieved inside the reaction chamber. Making use of these features, we established a multiplexed staining workflow (Fig. 1C). Initially, the slides are manually pre-processed for dewaxing and epitope retrieval (see details in Materials and Methods). Subsequently, the samples are sequentially stained and treated for antibody removal for 4 markers of interest from the breast cancer panel: estrogen receptor (ER), progesterone receptor (PR), epidermal growth factor receptor 2 (Her2) and cytokeratin (CK). Successful staining and antibody removal cycles take from 17 to 23 minutes per marker, reaching a total time of 1 h 24 min for a 4-plex staining (see below). Finally, the slides are removed from the stainer, coverslipped and scanned with a multi-spectral epifluorescence microscope.

The antibody removal characterization was designed as a 2 step protocol in which the detection is based on horseradish peroxidase (HRP)-labeled secondary antibodies that are tagged by a TSA-Alexa Fluor (AF) system. The characterization is performed using ER, PR, Her2 and CK markers that are stained on adjacent breast tissue micro array (TMA) sections. During the first staining cycle of the characterization, the markers of interests were specifically stained using TSA-AF647. Next, the antibody removal step was performed using the temperature control module that enables rapid heat transfer to the chamber. This step, carried out at 60 °C, removes the primary and secondary antibodies, leaving the deposited tyramide compounds covalently bound to the tissue^17^ (see temperature cycles in Fig. S1 Supplementary Information). Following the removal of the antibodies from the first cycle, we applied a second staining cycle, during which no primary antibody was applied to the tissue but only a detection system with TSA-AF488 (PBS/Ab-II/TSA AF488). This way, if any primary or secondary antibody is left after the antibody removal step, it could be observed on the AF488 channel. Equivalently, absence of signal in AF488 would imply that the primary and secondary antibody are indeed removed to an extent that it is not recognizable by subsequent staining cycles of a multiplexing assay.

After the 2-step protocol is applied to the 4 markers in question on adjacent tissue sections, we conducted a qualitative evaluation of the antibody removal efficiency determined by following two parameters: (i) preservation and integrity of the signal in the AF647 channel and its comparison with a monostaining and (ii) absence of the signal in the AF488 channel. In addition, these two parameters obtained by on-chip removal are compared with antibody removal using a standard MWT protocol (see the details of the MWT protocol in Materials and Methods).

**Figure 2:**
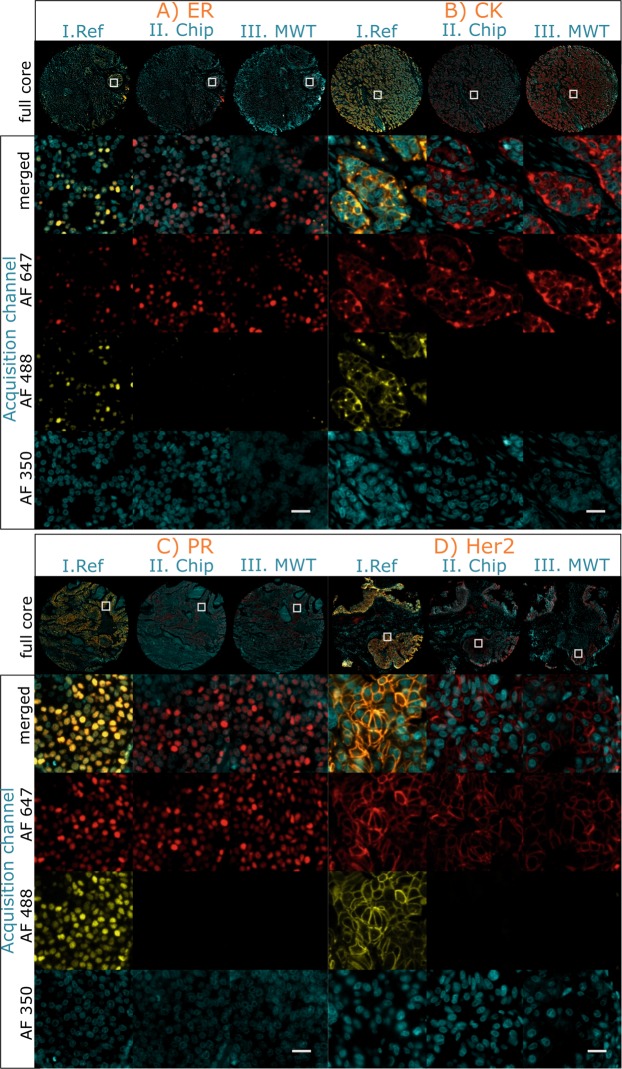
Characterization of the antibody removal efficiency in a 2-staining-cycle protocol. On-chip antibody removal efficiency is demonstrated for ER (**A**), CK (**B**), PR (**C**) and Her2 (**D**) and compared to the standard MWT. The characterization includes (i) a first detection using TSA-AF647 (Ab-I/Ab-II/TSA-AF647), (ii) heat-induced antibody removal, and (iii) staining with TSA-AF488 to detect the remaining primary and secondary antibodies (PBS/Ab-II/TSA-AF488). The figure shows the image acquisition in the AF647, AF488 and AF350 fluorescent channels, corresponding to a first staining cycle, a second staining cycle and the DAPI counterstain, respectively. (I. Ref) Experiment reference slide with no antibody removal step. All the markers are detected in both AF647 and AF488 channels. (II. Chip) On-chip antibody removal method: the markers are detected only in the AF647channel, while no fluorescent signal is detected in the AF488 channel. (III. MWT) Manual MWT: similarly, the markers are detected only on the AF647 channel. On-chip and MWT methods show equivalent outcome with respect to the evaluation criteria (see main text), however, fully automated on-chip antibody removal in 5 minutes eliminates the need of manual MWT treatment requiring 35 minutes. All the samples have been imaged with the same exposure settings and are visualized with the same parameters. Scale bar: 25 *μ*m.

Figure 2II shows the results of the 2 step characterization experiments. The antibody removal step performed on-chip resulted in a correct visualization of the markers revealed using TSA-AF647, while it was impossible to detect a signal on the TSA-AF488 channel, as expected. Figure 2III shows a comparison between our on-chip antibody removal method to the standard MWT protocol found in literature. Given the absence of signal in the AF488 channel, also in this case, the results indicates that both primary and secondary antibodies were removed by the MWT, while the AF647 tyramide compounds deposited on tissue were preserved. Additionally, as shown in Fig. 2I, a reference sample is stained by omitting the antibody removal steps (i.e. neither on-chip nor MWT) as an experiment control, in which the marker of interest is visible in the two acquisition channels: AF647 and AF488.

The results demonstrate that the on-chip and MWT antibody removal methods are comparably efficient under the above evaluation criteria. Consequently, the combination of the here proposed fast heat exchange in the small-volume reaction chamber and the on-chip protocol can perform an antibody removal within 5 minutes on the average, with respect to the 35 minutes required for MWT.

Additional control experiments, depicted in Table S1 and Fig. S2 of the Supplementary Information, were run with the scope of assessing the efficacy of the 2-step elution method. Three controls were run: (i) Ab-I/Ab-II/2-step elution/Ab-II/TSA-AF488/DAPI: Positive staining for PR, followed by elution, Ab-II and TSA-488. Negative results show that our elution method is efficient, here observed in the same channel as the positive staining of Fig. 2; (ii) Ab-I/PBS/2-step elution/Ab-II/TSA-AF488/DAPI: Positive staining for PR with no Ab-II, followed by elution, Ab-II and TSA-488. Results show no signal, indicating that our elution method is efficient in removing the primary antibody; (iii) Ab-I/Ab-II/single step at 60 °C /PBS/TSA-AF488/DAPI: Positive staining for PR, followed by a 60 °C step and TSA-488. Results show that PR can be detected and, therefore, a single step at 60 °C is neither enough to elute the antibodies nor to deactivate the HRP molecule.

Moreover, image analysis was performed on the data from Fig. 2 using the AF647 channel in order to quantify the signal levels for ER, CK, PR and Her2 before (AF647) and after (AF488) elution. Results demonstrate that the pixel grey values after elution measured on the signal masks are comparable to the grey values measured on the background masks (see details in Fig. S3 of the Supplementary Information). In terms of elution efficiency, the best case is observed for CK, with an elution efficiency of 99.36%, while the less performing marker turned out to be ER with 84.90%. The details of the calculations can be found on Table S2 of the Supplementary Information.

### On-chip 3-plex staining with antibodies originating from the same species

**Figure 3:**
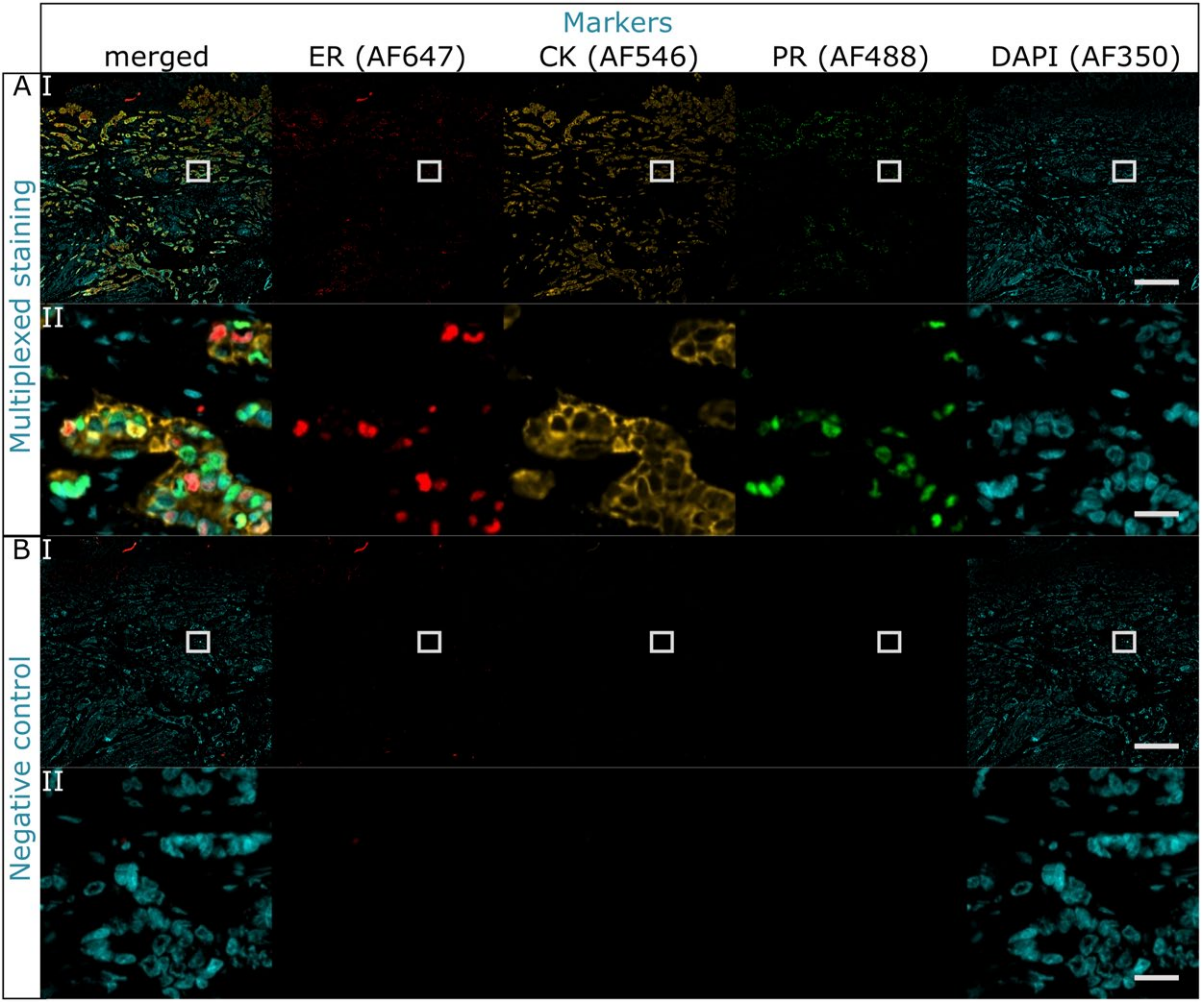
3-plex staining with primary antibodies originating from the same species obtained with the on-chip antibody removal method. (**A**) Multiplexed colocalized staining of ER, CK and PR. (**B**) Negative staining where the primary antibodies were replaced by washing buffer. All the markers were specifically stained and detected in their corresponding detection channel: AF647 for ER, AF546 for CK, AF488 for PR and AF350 for DAPI. No crosstalk between channels of subsequent stainings indicates the efficient removal of antibodies originating from the same species and no damage to the epitopes. For every panel: row I is the overview image, scale bar 500 *μ*m; row II is a zoom-in where the 3 markers are co-expressed, scale bar 25 *μ*m.

As a proof of concept, a colocalized 3-plex staining using antibodies from the same species (mouse) was successfully tested for ER, CK and PR. The markers were detected with the same secondary anti-mouse HRP-labeled antibody and TSA labeled with the fluorophores AF647, AF546 and AF488, respectively. Antibody removal is attained on-chip thanks to fast heat exchange described in the previous paragraph. Successful detection is observed without any crosstalk, indicating that the antibody removal method was efficient in a multiplexed staining and that the epitopes are not harmed in the process. The fast fluid exchange enabled the uniform delivery of reagents and coloration of the tissue, as shown in Fig. 3I for (A) the positive staining, and (B) its negative control. In the negative control, each primary antibody was replaced by washing buffer, while the incubation time and concentration of the secondary antibody and TSA are unaltered. Overall, the complete 3-plex staining protocol took 56 minutes and was run on-chip in a fully automated manner. Additional control experiments were ran to exclude that the presence of non-specific IgGs from mouse would lead to binding in our workflow. Results show equivalence between using PBS and non-specific IgGs diluted to the same mass concentration to the specific primary antibodies used for staining (see Tables S3, S4 and Fig. S4 of the Supplementary Information).

### Fully automated 4-plex staining of the breast cancer panel is achieved in 84 minutes

Image acquisition represents a limiting factor in the number of markers that can be multiplexed on a tissue sample using multi-spectral settings. In fact, the emission of each fluorophore must not overlap with the neighboring one. Typically, the number of filter sets that a standard fluorescence microscope can accommodate without spectral crosstalk in the visible range is 4^16^. In our case, the limitation is to 4 different channels corresponding to the fluorophores AF350, AF488, AF546/AF594 and AF647. Starting from the 3-plex proof of concept described above, we further pushed the multiplexing level to 4 markers by adding the detection of Her2. The antibodies employed for Her2 recognition originate from rabbit and are detected by a TSA labeled with AF350 fluorophore, imaged with the same filter set used for DAPI. To discriminate between the Her2 signal and the nuclei counterstain, we performed a control via a double-image approach, explained in details in the Materials and Methods.

**Figure 4:**
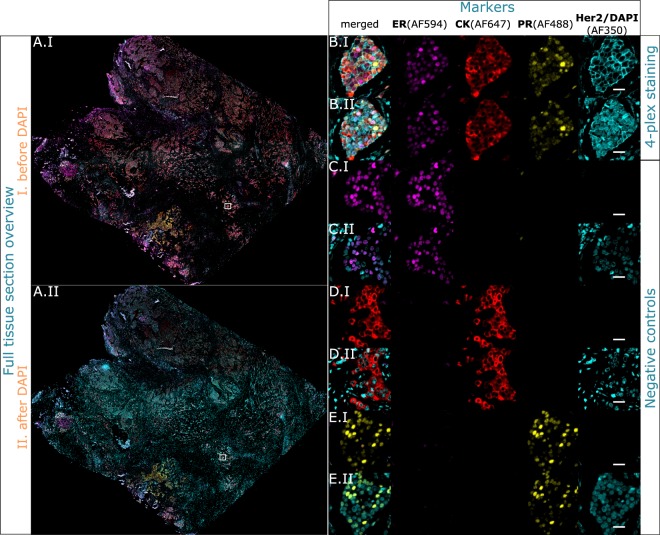
Multiplexed colocalized staining on-chip of ER (AF594), CK (AF647), PR (AF488) and Her2 (AF350) in 84 minutes. Full-tissue staining on a breast carcinoma sample for ER, CK, PR and Her2 is achieved in 84 minutes, excluding slide pre-processing. All the markers are specifically detected. Her2 and DAPI are visualized in the same channel (AF350) by imaging before and after counterstaining. (**A**) Overview image of the whole tissue section. (**B**–**E**) Zoom-in area where the 4 markers are co-expressed, visualized in the 4 acquisition channels. (**B**) 4-plex protocol. (**C**) Negative control where only ER is stained to be used as a focus reference. (**D**) Negative control where only CK is stained to be used as a focus reference. (**E**) Negative control where only PR is stained to be used as a focus reference. For every sequence panel: (I) is the first image acquisition without nuclei counterstain, (II) is the second acquisition after the nuclei were counterstained with DAPI. Scale bar 25 *μ*m.

Overall, the 4-plex protocol time took 1 h 24 min. Unlike the multiplexing protocols that use MWT for antibody removal, the on-chip protocol was fully automated with no need to handle the tissue sample in between staining cycles. The results shown in Fig. 4 indicate that the fluorescent signal is clearly distinguishable from the background. Additionally, the detection is specific for every marker and every core, with the sole exception of Her2 in Core B, where a weak non-specific signal can be observed. The insets of Fig. 4 focus on a tissue area where marker expression is expected.

The negative controls are performed by replacing the primary antibodies of the 4-plex protocol with washing buffer. As each negative control has to be imaged both prior and after the application of DAPI, negative controls prior to DAPI lacked a focus plane reference for the fluorescent microscope. To overcome this, we designed a set of 3 tissue sections as negative control, where in each control one marker was stained to serve as a focal reference, and the remaining 3 markers were negative controlled.

Figure 4B–D show the negative controls on adjacent sections for the markers ER, CK and PR, respectively, without DAPI (row I for every marker) and with DAPI counterstain (row II). The same acquisition and visualization settings of the 4-plex positive staining are maintained in the negative controls. The results show that every marker is specifically detected only in one channel, with no fluorescent signal in the others.

**Figure 5:**
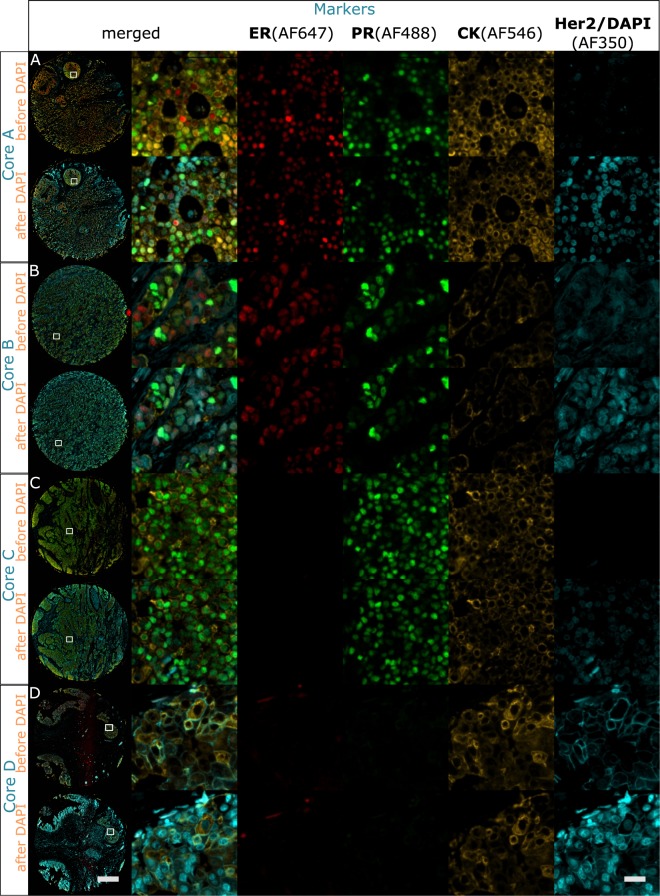
Colocalized 4-plex staining of ER (AF647), CK (AF546), PR (AF488) and Her2 (AF350) applied to the breast TMA. 4-plex staining performed on the same cores used for the antibody removal characterization in Fig. 2. Specific staining is observed for each marker. The left column shows the overview of the TMA cores, scale bar 500 *μ*m. Columns 2–6: (**A**) Core employed for the antibody removal characterization of ER (Fig. 2A), expressing also PR and CK staining and negative to Her2. (**B**) Core employed for the antibody removal characterization of CK (Fig. 2B), expressing also ER and PR, and negative to Her2. (**C**) Core employed for the antibody removal characterization of PR (Fig. 2C), expressing also CK and negative to ER and Her2. (**D**) Core employed for the antibody removal characterization of Her2 (Fig. 2D), expressing also CK and negative to ER and PR. Scale bar 25 *μ*m.

In order to assess the specificity of the 4-plex protocol with respect to single staining, the above-introduced method and imaging approach were used to run the colocalized 4-plex protocol on a breast TMA, and the expression results are compared with monoplex stainings. Figure 5 shows the results of the double image acquisition. For every marker of the TMA, the intensity of the microscope acquisition filters and the exposure time have been tailored on the brightest core and applied to the entire TMA. For each core, we observed that the expression of every marker stained in the 4-plex protocol (Fig. 5) matches its expression in the single staining control (Fig. 2). Consequently, for all the 4 markers, we have found no specificity difference between the 4-plex protocol and the single staining controls. Note that only 4 out of 10 TMA-cores corresponding to the same cores employed for the antibody removal characterization are shown in the Fig. 5, for the entire TMA visualization, see Fig. S5 of the Supplementary Information). Additionally, Table S5 of the Supplementary Information shows the TMA core description given by the supplier. IHC staining using Ventana’s Benchmark Ultra was also performed at the University Hospital of Lausanne. The overview IHC images of the TMA are reported in Fig. S6, where the general expression and tissue morphology can be appreciated and compared to the fluorescence assays shown in this work.

## Discussion

We show an on-chip multiplexed immunostaining method that enables the colocalized staining of ER, CK, PR and Her2 on human tissue sections with antibodies originating from the same species in a record time of 1 h 24 min. Two major advantages over other works are presented here: i) the rapidity of the staining and the antibody removal method and ii) the full automation of the protocol. The presented microfluidic technology allows to precisely control multiple parameters needed for the staining, mainly the uniform delivery of reagents and the temperature inside the reaction chamber. Plus, its high surface-to-volume ratio allows a rapid heat transfer to the thin fluid layer thus reducing the heating and cooling stabilization time for the sample. Overall, the sample processing time is reduced from several hours to few minutes. The incubation time of the primary antibodies for cytoplasmic and membrane markers is only 2 minutes, while for the nuclear markers 4 minutes are required. In comparison, fully automated state-of-the art workflow automation systems operate in the range of 60 minutes^16–18^.

The multiplexed protocol is accompanied by single reference stainings as well as negative controls, to assess (i) efficiency of antibody removal, (ii) the specificity of the detection, and (iii) to determine the markers’ expression profile. Despite the divergent opinions regarding the most efficient antibody stripping method^7,14,15^, microwave treatment is often employed to enable multiple stainings with antibodies originating from the same species and TSA detection^16,17,21,23^. We employed MWT as a reference antibody removal method to compare to our fast, automated on-chip antibody removal within 5 minutes. For every marker, our protocol involves a high temperature step at 60 °C for 2 minutes, necessitating only a stabilization time of few seconds due to the fast heat exchange inside the reaction chamber. In contrast, in similar studies demonstrating antibody removal based on thermal cycles, only reaching the desired temperature needs around 8 minutes^17^. To further ensure that also the antibodies are removed in such short duration, following the removal step, we incubated the sections again with HRP secondary antibody and detected by a TSA labeled with a different fluorophore. The preservation of the signal in the staining channel and absence of the signal in the after-removal channel proved the efficiency of our removal method.

The microfluidic design ensured the uniform distribution of the reagents over the entire section and enabled a colocalized staining throughout the tissue. Recently, an AC electric field enhancement device to stain frozen tissue sections demonstrated comparably fast staining, however it was only tested for a single antibody (CK)^24,25^. Spatial multiplexing devices with staining times in the order of 1 hour could not provide colocalization^9,10,26^. Our fully automated system and multiplexing protocol, on the other hand, is currently capable of staining a tissue sections and TMAs as large as 17 × 17 mm (Fig. 4). Additionally, the microfluidic technology is scalable to any chamber size. The signal for nuclear, cytoplasmic and membrane markers on the 4-plex assay, is comparable to the single positive controls. While the whole tissue sample was chosen to highly express all the markers of interest, specificity was addressed relying on the multiple tissues composing the TMA.

The here presented technology requires an average processing time of 20 minutes per marker, including all washing cycles. To our knowledge, this is the first time that a colocalized 4-plex staining is run fully automated in only 84 minutes, a shorter time than typically required for a full IHC staining with standard automated processes. Overall, our platform offers the possibility to explore multiple parameters in record time, which is essential in the frame of application development and impossible to accomplish with other devices in comparable time. It dramatically reduces the assay development time, for example, when (a) optimizing the clone and the concentration of the antibody, (b) identifying sequence of primary antibodies in the multiplexing protocol, and (c) for coupling low expressing markers with brighter fluorophores. Therefore, while we demonstrated our method on breast cancer markers here, it is transferable to research domains, such as immunophenotyping, where multiplexing is extremely beneficial to understand the tumor microenvironment. Furthermore, our characterization showing equivalence of microfluidic on-chip antibody removal to MWT antibody removal implies that the presented 4-plex method shall easily be extendable to multiplexing of higher number of tissue biomarkers^16–18,27^. In addition, since potential tissue-antigen degrading effects of the heat-induced antibody removal period is reduced to 2 minutes, one would expect that fast heat exchange will possibly allow higher-plexing compared to MWT or other methods.

Finally, at present, the presented method constitutes a very powerful device for research and assay development, offering unrivaled time-to-result, which would drastically reduce assay development times. In addition, the full automation of the device eliminates the variability caused by the user intervention. It is clear that further work, including but not limited to image analysis, quantification of the antibody removal and demonstration of higher level of multiplexing, should be carried out before transferring this ultra-fast technology as a routine tool. Nevertheless, the reported duration with full automation opens up avenues for the integration of the multiplexed immunostainings in the routine practice in highly needed applications like small tissue samples or immunophenotyping. We are confident that in the future our technology can contribute widespreading of -omics like approaches in tissue diagnostics.

## Methods

### Microfluidic technology for fast fluidic exchange and rapid heat transfer

Based on the MTP device previously described^19,20^, the system was further engineered to make the staining procedure fully automated and reproducible independently from the user. The MTP chip is clamped with a microscope glass slide, on which the tissue section is fixed, in order to create a reaction chamber with a thickness of 100 *μ*m (Fig. 1). A polydimethylsiloxane (PDMS) gasket defines the height of the chamber and ensures its proper sealing. The height of the chamber is one of the most critical parameters and guarantees a fast and uniform distribution of the reagents over the staining area (17 × 17 mm). Two toggle clamps linked to a cover lid and a pressure-controlled piston close the reaction chamber. An inlet branch of microfluidic channels connects the reaction chamber to a reagent delivery system, while three outlet branches address the reagents into a waste container. The reagent delivery system, depicted in Fig. S7 of the Supplementary Infromation, allows to select the reagent to dispense from a pool of 8 vials and 4 falcons through a pressurized valve system that automatically controls the valve opening and closing. Below the MTP chip, a Peltier element (Laird Technologies SH10, 125, 05, L1, W4.5), supported by a forced-air cooling system, allows to control the temperature and the heat transfer inside the chamber. The feedback signal coming from a resistance thermometer (Heraeus PT100 FK222) in contact with the MTP surface is used to monitor the temperature inside the chamber and to regulate the current supplying the Peltier. This configuration allows a uniform and fast heat transfer to the thin layer of liquid inside the chamber.

### Tissue preparation and reagents

Breast tissue samples were purchased from EastWestBiopharma (Kyiv, Ukraine) upon Service Agreement for Collection of Human Biological Material and Associated Data. The TMA samples were purchased from US Biomax, Inc. (MD, USA) upon statement consent form and feature 10 breast cores with different combinations of markers’ expression. The full tissue sections were selected in order to have the 4 markers of interest, i.e. ER, CK, PR and Her2, co-expressed on the same tissue case. Both were provided as 4 *μ*m FFPE sections mounted on Superfrost Plus slides (Thermo Scientific). Sample preparation was performed manually off-machine. Tissue samples and TMAs were at first dehydrated 10 minutes at 65 °C, dewaxed using Histoclear (National Diagnostics, GA, USA) for 10 min and then rehydrated using ethanol solutions in decreasing concentrations (100%, 95%, 70% and 40% vol/vol) (Fisher Chemical) for 2 minutes. Afterwards, heat-induced antigen retrieval (AR) using Tris/EDTA buffer pH9 (Dako S2367, Denmark) was run for 40 minutes at 95 °C in a hot bath, followed by 20 min cooling at room temperature. Then, the samples were immersed in phosphate buffered saline pH 7.4 (PBS) (Sigma Aldrich, MO, USA) before being loaded on the stainer device.

As primary antibodies the following reagents were employed: mouse anti-human cytokeratin, clone AE1/AE3 (code M3515, Dako, Denmark) at the concentration of 1.72 *μ*g/ml, mouse anti-human progesterone receptor (Novocastra NCL-L-PGR-312) at the concentration of 42 *μ*g/ml, mouse anti-human estrogen receptor (Novocastra NCL-L-ER-6F11) at the concentration of 0.7 *μ*g/ml, rabbit anti-human c-erbB-2 oncoprotein (A0485, Dako, Denmark) at the concentration of 2.4 *μ*g/ml. The detection was performed using ready-to-use secondary anti-mouse or anti-rabbit HRP-labeled antibodies (ImmPRESS, Vector Laboratories MP-7402 and MP-7401, respectively) together with one of the following AF-coupled TSA (Life Technologies, CA, USA): AF350 (B40952), AF488 (T20948), AF546 (T20913), AF594 (T20950), AF647 (T20951). All the reagents were dissolved in a 0.05% (vol/vol) solution of Tween 20 (BP337-100, Fisher Scientific, MA, USA) in PBS, except TSA-AF350 that was dissolved in a 0.05% (vol/vol) solution of Tween 20 in Tris Buffered Saline (TBST) (BP2471-1 Fisher Scientific, MA, USA). The TSA reagents were activated by adding 0.0015% H_2_O_2_ (216763, Sigma Aldrich, MO, USA). In the case of 4-plex staining, a water-based mounting solution SlowFade Gold Antifade (S36936, Thermo Fisher Scientific, MA, USA) was used to mount the slides without nuclear counterstain. For all the others, SlowFade Gold Antifade with DAPI (S36938, Thermo Fisher Scientific, MA, USA) was employed to mount the coverslips and counterstain the nuclei. Coverslips no. 1 from Knittel Glass are employed.

### Automated staining protocol

Once the glass slide is loaded on the stainer, the reaction chamber is closed by applying 2.5 bar pressure to the piston supporting the MTP chip. The staining protocol for CK and Her2 is 10 minutes in total, as reported in details in Table 1 (steps 5–7 and steps 13–15, respectively). The staining protocol of the nuclear markers ER and PR requires overall 14 minutes (steps 1–3 and steps 9–11, respectively). PBS buffer was used as washing buffer in between the staining steps and delivered at 25 *μ*l/s for 10 s. Primary and secondary antibodies have been incubated for 2 minutes for CK and Her2, and for 4 minutes for ER and PR. TSA was incubated for 2 minutes in all the experiments. The rationale behind the choice of the concentration of the reagents and their incubation times have been already discussed in previous works^19,20^. All the reagents were delivered at 25 *μ*l/s for 8 s. Upon completion of the staining protocol on-chip, the sample slide is unloaded from the device and manually rinsed with deionized water for few seconds before being mounted with a coverslip using the mounting solution.

**Table 1:** 4-plex protocol applied to the MTP stainer.

Step	Reagent	Incubation time min
1	anti-ER AbI	4
2	HRP-AbII	4
3	TSA-AF	2
4	Elution	6
5	anti-CK AbI	2
6	HRP-AbII	2
7	TSA-AF	2
8	Elution	4
9	anti-PR AbI	4
10	HRP-AbII	4
11	TSA-AF	2
12	Elution	6
13	anti-Her2 AbI	2
14	HRP-AbII	2
15	TSA-AF	2
	Total staining time	48 min
	Total staining time with washing steps	1h24 min

TSA-AF indicates a fluorescent-labeled TSA among AF350, AF488, AF546, AF594 and AF647.

#### Characterization of antibody removal

A double staining on the same tissue sample is used to characterize the efficiency of the antibody removal method. The first staining cycle consists in the positive staining of one marker (ER, PR, CK or Her2) detected by TSA-AF647. The second one consists in a negative step where the primary antibodies are replaced by a PBS incubation of equivalent time and the detection relies on TSA-AF488. In between the first and the second cycles, antibody removal is performed. Based on qualitative visual inspection, the antibody removal is considered efficient when the marker of interest is detected only with TSA-AF647 and not with TSA-AF488. The antibody removal on-chip consists in a first exposure to buffer I at room temperature for 2 minutes, in the case of CK and Her2, and for 4 minutes, in the case of ER and PR. Afterwards, a sequent incubation with buffer II at 60 °C for 2 minutes is applied for all the markers. This step is immediately followed by the second staining cycle at room temperature. Overall, the protocol takes 17 minutes for CK and Her2, and 23 minutes for ER and PR. Buffer I contains sodium dodecyl sulfate and buffer II contains tris(hydroxymethyl)aminomethane and ethylenediaminetetraacetic acid. The antibody removal off-chip is performed with MWT. The slides are immersed in a jar containing AR pH9 and placed inside the microwave. The microwave is run 50 s at full power (100%), followed by 15 minutes at 20% power. Afterwards, the slides are left 20 minutes immersed in the AR for natural cooling and are loaded again on the MTP stainer for the second staining cycle. The reference control slide does not undergo any antibody removal process, it is incubated 2 minutes on-chip with PBS washing buffer. No removal of antibodies is applied leaving the primary and secondary antibodies on the tissue surface, that can therefore be detected also by TSA-AF488 during the second staining cycle.

#### Multiplexing staining protocol with antibodies from the same species

A full 3-plex protocol for the staining of ER, CK and PR, in this order, is run on the MTP stainer with the antibodies removal method on-chip (Table 1, steps 1–11).

The protocol takes 54 minutes, at the end of which the slide is manually rinsed with DIW and mounted with a coverslip using the mounting solution with DAPI for nuclear counterstain. The image is acquired only at the end of the experiment for all the channels. The negative control is performed by replacing the primary antibodies with PBS washing buffer of equivalent incubation times.

#### 4-plex automated staining protocol

A 4-plex protocol for the staining of ER, CK, PR and Her2 in this order, is run on the MTP stainer with the antibody removal method on-chip (Table 1). Her2 is detected with TSA-AF350, which is excited and emits at the same wavelengths of DAPI, employed to counterstain the nuclei. Therefore, a double-image approach is introduced to discriminate the signal coming from Her2 and the one coming from DAPI. At the completion of the 4-plex protocol, a cover-slip is mounted using a water-based mounting solution that does not contain DAPI. After the image is acquired, the coverslip is unmounted: the sample slide in immersed 10 s in deionized water and the coverslip is removed with the help of tweezers. Then, the slide is manually rinsed with deionized water and mounted with a new coverslip using the mounting solution containing DAPI. Thus, there are two images of the same slide, one without nuclei counterstained and one with the nuclei counterstained with DAPI. Because it is not possible to focus during the image acquisition in absence of DAPI, the negative controls are performed as a single staining of one marker in a 4-plex negative protocol for ER, CK and PR. The order of incubation is conserved (ER-PBS-PBS-PBS, PBS-CK-PBS-PBS, PBS-PBS-PR-PBS) and the same concentration and incubation time are applied.

#### Fluorescence image acquisition

The slides were loaded into an automated epifluorescent microscope (Axio Imager M2, Zeiss, Germany) and a CCD camera was employed to acquire mosaic images. Zeiss filter sets 02, 10, 43HE, 64HE, 50 were employed for the AF350, AF488, AF546, AF594 and AF647 fluorophores, respectively. The filter intensity and the exposure time adopted are reported in Table 2. Acquisition, scanning and stitching were done automatically. The autofocus was set in the AF350 channel and run automatically for every tile of the image, a correction of −2.3 *μ*m was applied when acquiring in the AF647 channel upon focus offset characterization. The overall acquisition time varies between 10 to 50 minutes, depending on the size of the sample and on the number of channels employed.

**Table 2:** Settings applied for the microscope image acquisition.

Fluorophore	Filter	Light intensity (%)	Exposure time (ms)
AF350	02	2	200
AF488	10	2	180
AF546	43HE	2	100
AF594	64HE	20	50
AF647	50	100	300

## Supplementary information

Supplementary Information

## Data Availability

The datasets generated during the current study are available from the corresponding author on reasonable request.
