# Supplementary material for: Ultra-fast and automated immunohistofluorescent multistaining using a microfluidic tissue processor
Source: Sci Rep. 2019 Mar 14;9:4489. doi: 10.1038/s41598-019-41119-y (PMC6418167; doi:10.1038/s41598-019-41119-y)
Supplement: Supplementary file 1 — Supplementary Information [file 41598_2019_41119_MOESM1_ESM.docx]

Supplementary Information

**Ultra-fast and automated immunohistofluorescent multistaining using a microfluidic tissue processor**

**Authors:** Giulia Cappi, Diego Gabriel Dupouy*, Marta Aurelia Comino and Ata Tuna Ciftlik

**Affiliation:**

Lunaphore Technologies SA, EPFL Innovation Park, Building C, 1015 Lausanne, Switzerland

*Corresponding author. e-mail: diego.dupouy@lunaphore.com

**List of Figures:**

Figure S1. 4-plex temperature profile

Figure S2. Elution control experiments.

Figure S3. Signal quantification with and without elution.

Figure S4. Negative controls comparison.

Figure S5. Results of the monoplex characterization and 4-plex staining for all the cores of the TMA

Figure S6. IHC reference staining.

Figure S7 Reagent Delivery System (RDS).

**List of Tables**

Table S1. Protocol steps for elution control stainings.

Table S2. Elution efficiency

Table S3. IgG dilutions for negative control staining comparison

Table S4. Protocol steps for positive and relative negative control stainings (single-plex

Table S5. TMA core description from supplier.


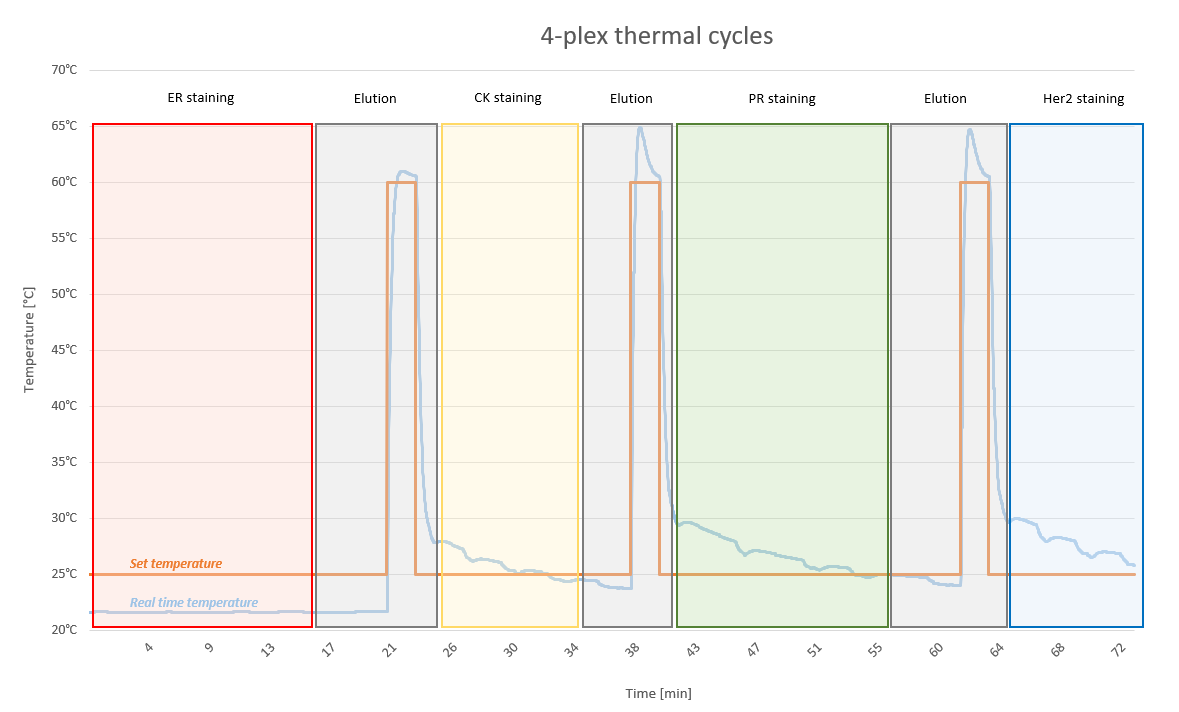


**Figure S1 4-plex temperature profile.** Set temperature (orange) and measured temperature (blue) during the staining and elution cycles performed for a 4-plex experiment of ER, CK, PR and Her2 in that order. Initial deparaffinization and antigen retrieval is performed manually off-chip.

**Control experiments on elution steps**

| Control | Sequence | | | | | | Scope of the control |
| --- | --- | --- | --- | --- | --- | --- | --- |
| 1 | Ab I | Ab II | **Elution** | Ab II | TSA | DAPI | This control shows the elution effect on the same TSA channel as the main staining, as opposed to the controls run in the main text using a second channel to observe the efficiency of the elution. |
| 2 | Ab I | PBS | **Elution** | Ab II | TSA | DAPI | The control shows that the elution we execute on chip is not quenching the HRP, but rather removing the antibodies. Indeed, after the elution step of Ab I (that does not present any HRP attached), the staining does not work, no signal is detected in the TSA channel. |
| 3 | Ab I | Ab II | 60°C step | PBS | TSA | DAPI | This control demonstrates that the step at 60°C does not affect the functionality of the HRP attached to Ab II. The presence of signal means that even after the exposure at temperature, the HRP still reacts with TSA. |

**Table S1. Protocol steps for elution control stainings.**

| Control # | Overview | ROI Merged / AF488 / DAPI |
| --- | --- | --- |
| 1 | 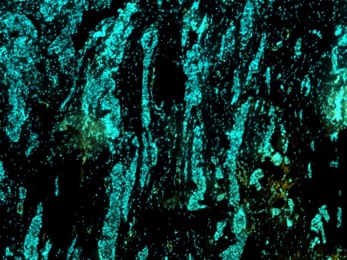 | 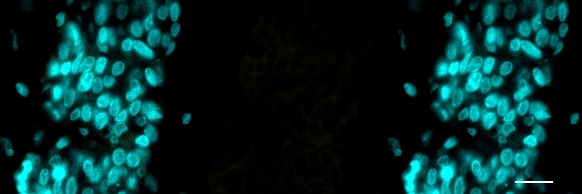 |
| 2 | 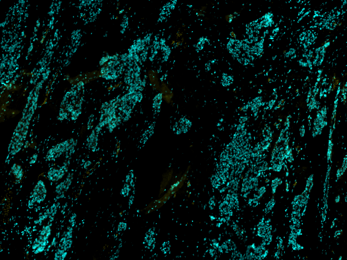 | 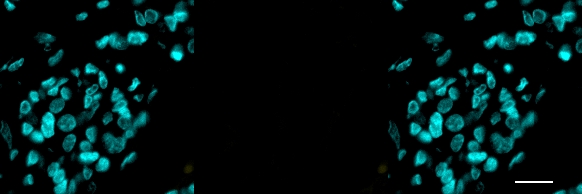 |
| 3 | 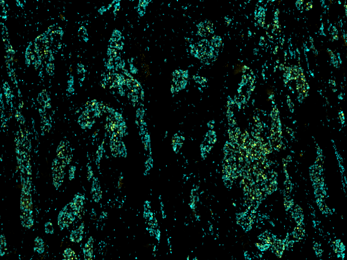 | 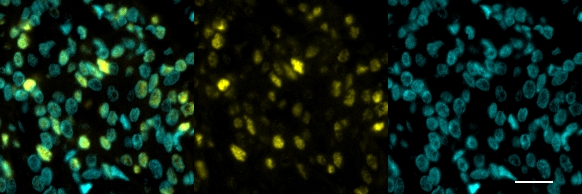 |

**Figure S2. Elution control experiments.**


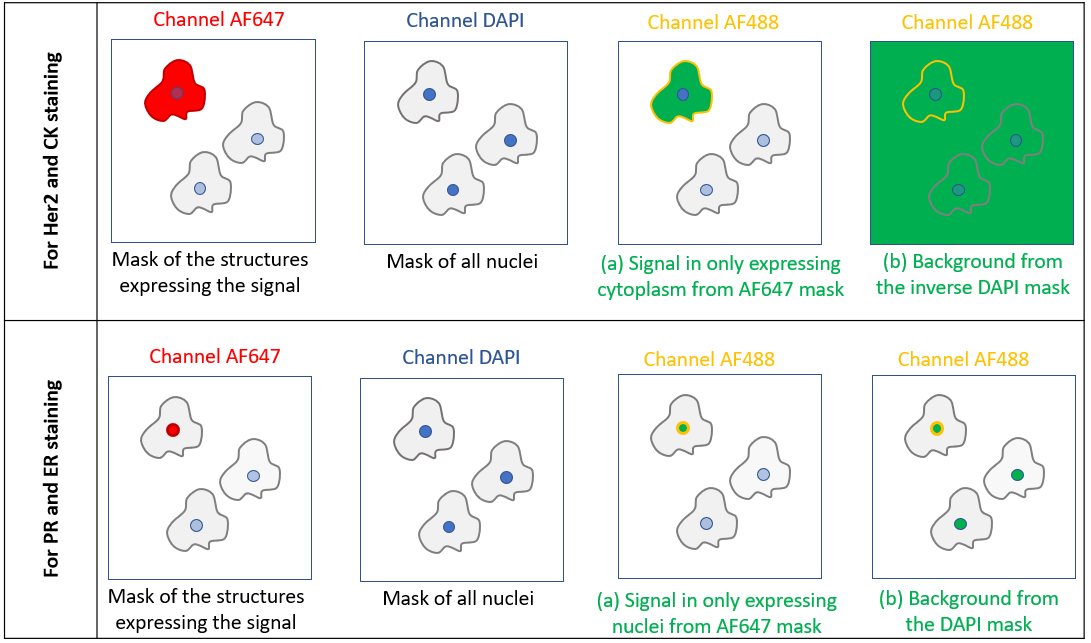
**Figure S3 Signal quantification with and without elution.** Elution (EL) efficiency is demonstrated on the signal level from the data shown on Figure 5 for Her2, CK, PR and ER on the AF488 channel. Intensity of detected signal after EL (blue dots) and intensity of the background after EL (orange dots), normalized to the non-eluted signal (grey dots) are plotted for on-chip EL (A) and microwave EL (B). (C) Image processing algorithm for signal and background intensity calculation. The signal after EL is calculated only on the structures of interest (a): cytoplasm and membrane for CK and Her2 (top panel) and nuclei for PR and ER (bottom panel). The background (b) is calculated based on a mask created from the DAPI channel.

C

B

A

|  | On-chip | MWT |
| --- | --- | --- |
| Her2 | 91,92% | 93,44% |
| CK | 99,36% | 97,03% |
| PR | 102,18% | 101,61% |
| ER | 84,90% | 91,13% |
| $Eq. S1: {EL}_{efficiency}=\left( 1-\frac{Signal after EL-Background}{Signal with NO EL- Background} \right)x 100\%$ | | |

**Table S2. Elution efficiency.** Elution efficiency calculated as shown in Eq. S1. The elution efficiency showed comparable results between both on-chip and microwave (MWT) methods, excepts for ER where the MWT elution showed to be more performant.

**Negative controls: equivalence between the use of PBS and IgG in a negative staining is demonstrate for two IgG dilutions.**

| **IgG mouse** | **Reference marker** | **IgG Dilution** |
| --- | --- | --- |
| Invitrogen mouse IgG Isotype Control  Product #02-6502  Vial Concentration: 2.5 mg/ml | Novocastra monoclonal mouse Progesterone receptor  Product #NCL-L-PGR-312  Concentration: 360 mg/l | Dilution (2500mg/l) / (360 mg/l) = 6.9x  1 µL of IgG concentrate in 5.9 µl of PBS |
| Invitrogen mouse IgG Isotype Control  Product #02-6502  Vial Concentration: 2.5 mg/ml | Dako monoclonal mouse anti-human Cytokeratin AE1/AE3  Product #M3515  Concentration: 200.8 mg/l | Dilution (2500mg/L) / (200.8 mg/l) = 12.45x  1 µl of IgG concentrate in 11.45 µl of PBS |

**Table S3.** **IgG dilutions for negative control staining comparison**. The IgGs used for negative control experiments were diluted to reach the same concentration as the corresponding antibody used for the staining experiment: 360mg/l for PR and 200.8 mg/l for Cytokeratins; PBS: Phosphate Buffer Saline.

| **Exp** | **Staining** | **Primary antibody** | **Secondary antibody** | **TSA** | **DAPI** |
| --- | --- | --- | --- | --- | --- |
| I. | PR – TSA488 | 4 minutes | 4 minutes | 2 minutes | 1 minute |
| II. | PBS – TSA488 | 4 minutes | 4 minutes | 2 minutes | 1 minute |
| III. | IgG - TSA488 | 4 minutes | 4 minutes | 2 minutes | 1 minute |
| IV. | CK – TSA647 | 2 minutes | 2 minutes | 2 minutes | 1 minute |
| V. | PBS – TSA647 | 2 minutes | 2 minutes | 2 minutes | 1 minute |
| VI. | IgG – TSA647 | 2 minutes | 2 minutes | 2 minutes | 1 minute |

**Table S4. Protocol steps for positive and relative negative control stainings (single-plex).** Duration of the main steps for monoplex stainings experiments and controls are detailed: incubation of primary antibody, secondary antibody, TSA and DAPI.

|  | Overview | ROI Merged / AF488 / DAPI |
| --- | --- | --- |
| I. PR staining | 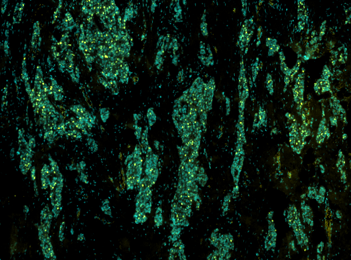 | 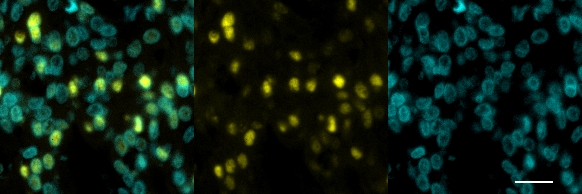 |
| II. Negative control with PBS | 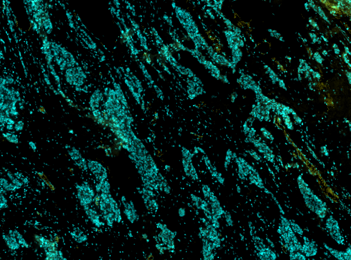 | 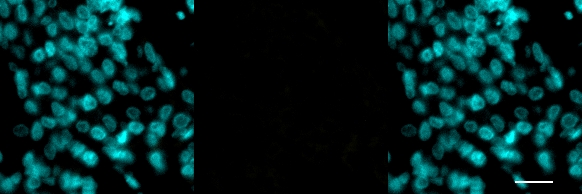 |
| III. Negative control with IgG | 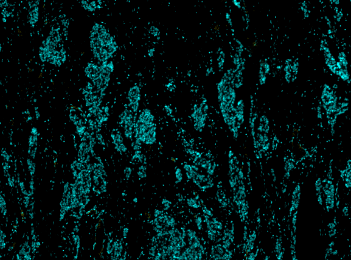 | 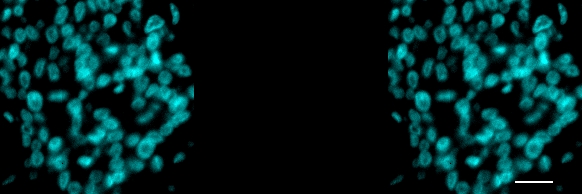 |
|  |  |  |
|  | Overview | ROI Merged / AF647 / DAPI |
| IV. CK staining | 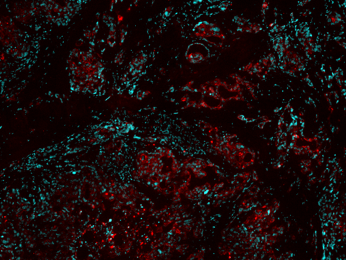 | 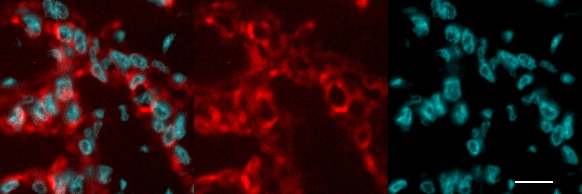 |
| V. Negative control with PBS | 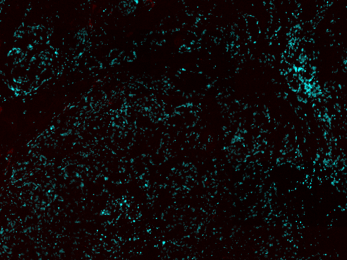 | 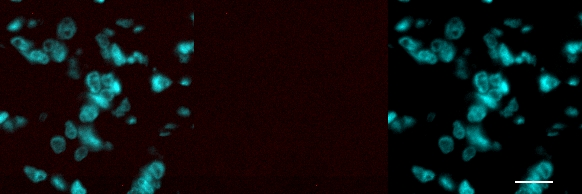 |
| VI. Negative control with IgG | 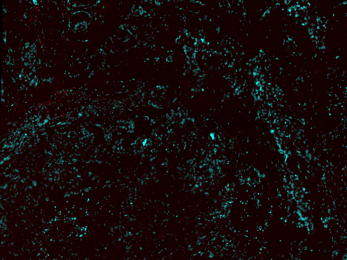 | 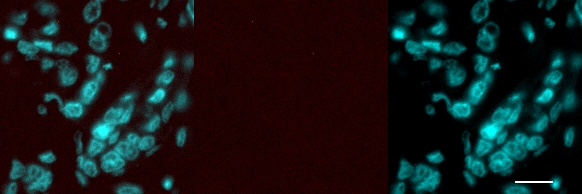 |

**Figure S4. Negative controls comparison.** Equivalence between the use of PBS and the IgG mouse diluted to the same concentration as PR and Cytokeratin, respectively, is demonstrate. Both controls result negative in the AF488 channel (II and III) compared to the PR staining (I) and in the AF647 channel (V and VI) compared to the Cytokeratin staining (IV). All images are visualized with the same brightness and contrast parameters. Scale bar 25 µm.


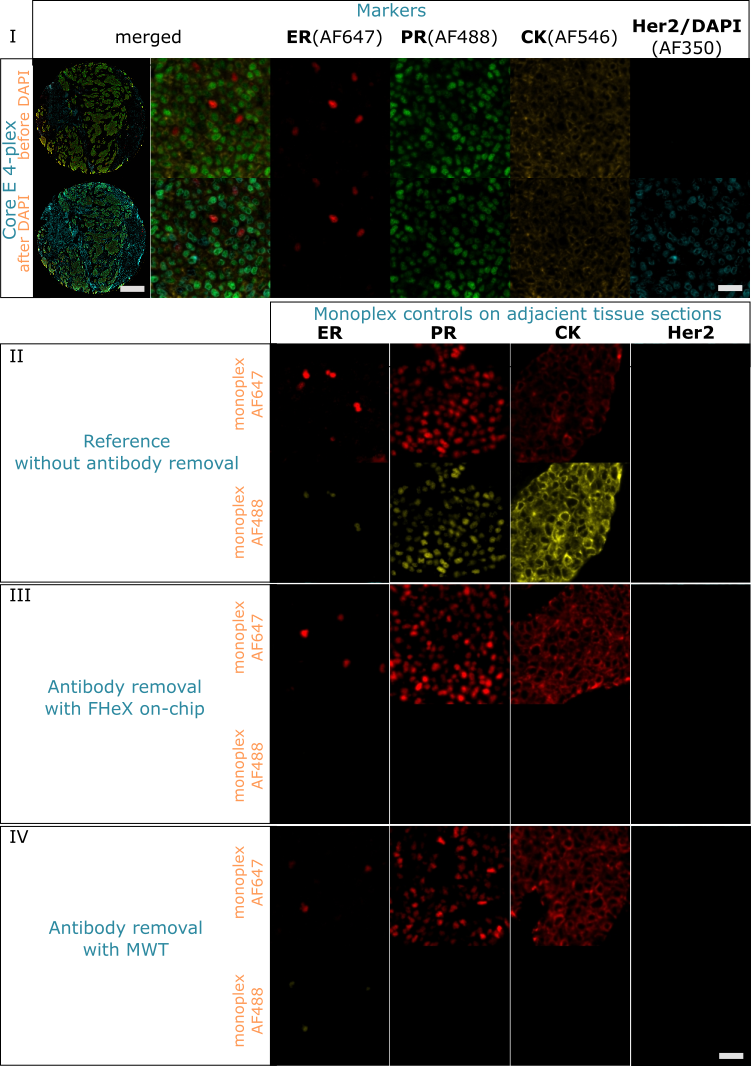


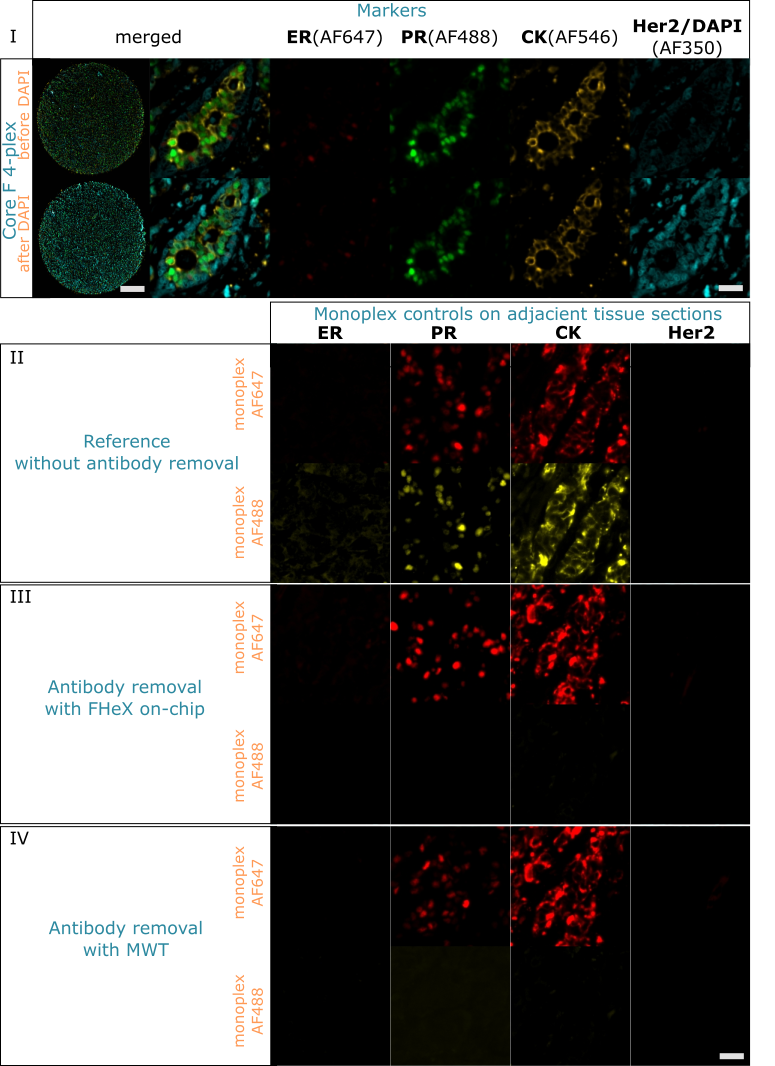


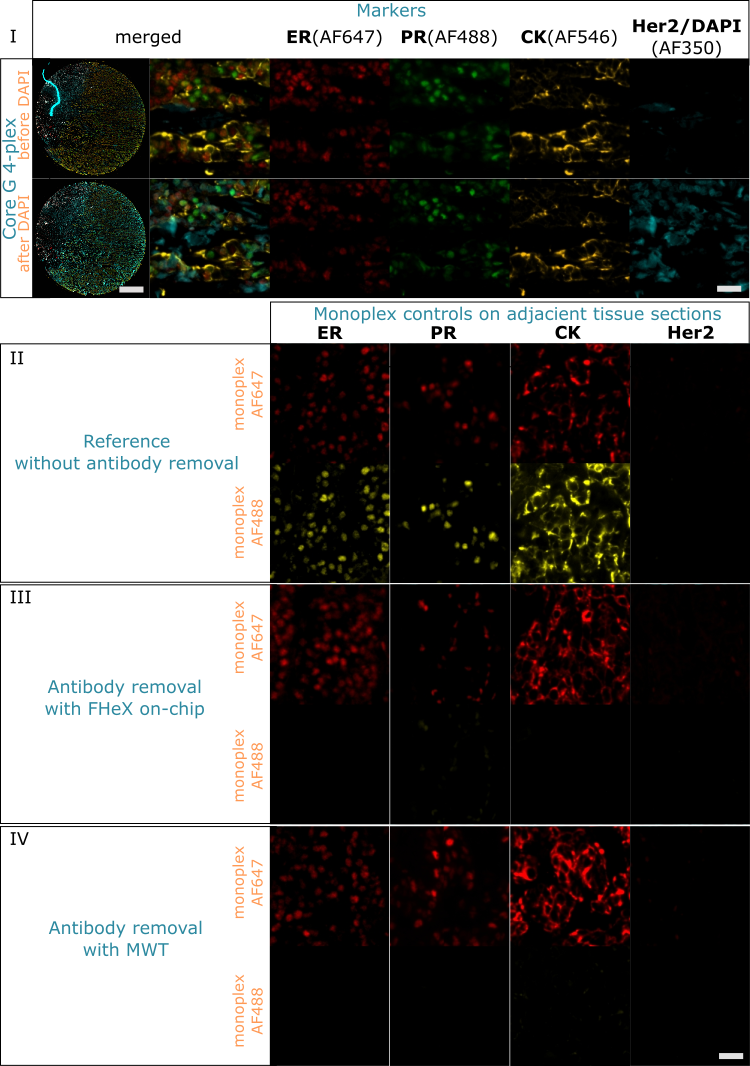


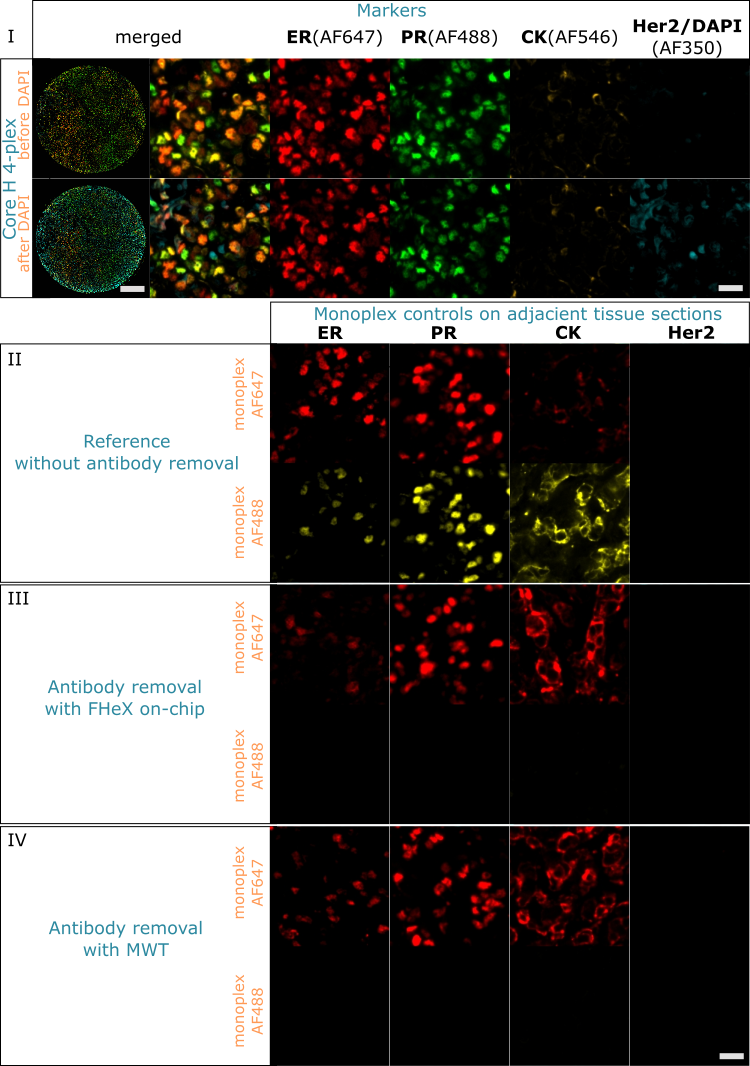


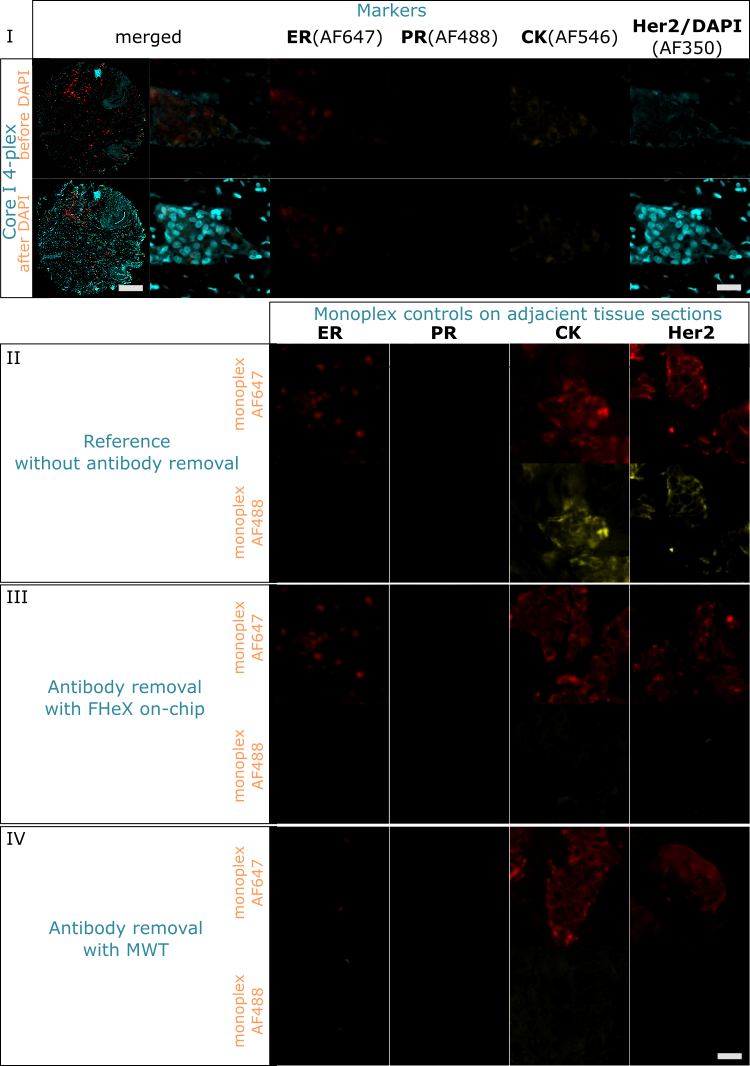


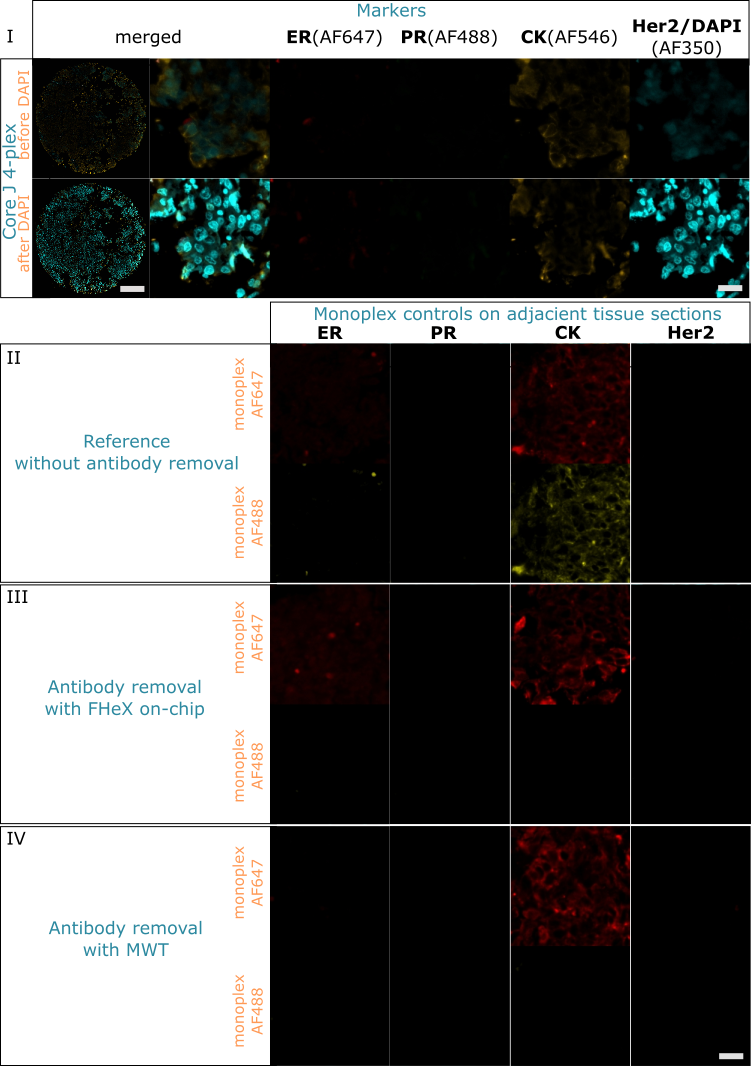


**Figure S5. Results of the monoplex characterization and 4-plex staining for all the cores of the TMA.** The TMA was employed to address the specificity of the elution method. The 10 cores composing the TMA present different expression of the markers ER, PR, CK and Her2.

For every panel of figure S1 (cores E-J):

1. shows the 4-plex protocol run on-chip in 84 minutes. The double-image approach is applied, therefore, Her2 and DAPI are visualized in the same channel (AF350) showing the signal before the application of DAPI (top line) and after the DAPI counterstaining (bottom line). ER, PR and CK are imaged in the channels AF647, AF488 and AF546, respectively, with the same acquisition and visualization settings employed in figure 5.
2. represents the control experiment of the 2-staining-cycle protocol used for the characterization of the antibody removal (figure 2, column I). The characterization experiment consists in a first staining of the marker of interest detected with TSA-AF647, followed by a second staining cycle without primary antibodies detected with TSA-AF488. In this control, the antibody removal step is omitted, making the marker of interest visible in the two acquisition channels: AF647 and AF488.
3. demonstrates the antibody removal on-chip when the FHeX is applied and corresponds to column II of figure 2 of the characterization experiment. During the FHeX, the primary and secondary antibodies applied during the first staining cycle are removed, leaving only the tyramide reaction compounds AF647 deposited on tissue. Thus, when the second staining cycle with TSA-AF488 is applied, the absence of primary and secondary antibodies results in the absence of detectable signal.
4. compares the antibody removal on-chip to the MWT (figure 2, column III). The primary and secondary antibodies applied during the first staining cycle are removed using manual MWT, leaving only the tyramide reaction compounds AF647 deposited on tissue. Thus, when the second staining cycle with TSA-AF488 is applied, the absence of primary and secondary antibodies results in the absence of detectable signal.

The characterization experiments were conducted on adjacent TMA sections by staining only one marker per slide and they are here employed to prove the specificity of the signal obtained in the 4-plex protocol. The image acquisition parameters were tailored on the brightest core and applied to the whole TMA. All the cores are visualized with the same brightness and contrast. Scale bar 25 µm.

| **Position** | **Core**  **(in text)** | **Sex** | **Age** | **TMA supplier information** | | | | | | |
| --- | --- | --- | --- | --- | --- | --- | --- | --- | --- | --- |
|  |  |  |  | **Organ** | **Pathology**  **Diagnosis** | **Grade** | **Stage** | **TNM** | **PR** | **Her2** |
| A1 | Not shown | F | 55 | Liver | Hepatocellular liver cancer | 3 | IIIa | T3N0M0 | - | - |
| A2 | Not shown | - | - | Blank | Blank | - | - | - | - | - |
| A3 | C | F | 35 | Breast | Invasive ductal carcinoma | 1 | IIIb | T4N2M0 | 85% | - |
| A4 | A | F | 71 | Breast | Invasive ductal carcinoma | 2 | IIa | T2N0M0 | 75% | ++ |
| B1 | B | F | 56 | Breast | Invasive ductal carcinoma | 1 | IIb | T2N1M0 | 50% | - |
| B2 | E | F | 47 | Breast | Invasive ductal carcinoma | 1 | IIb | T3N0M0 | 90% | - |
| B3 | F | F | 48 | Breast | Invasive ductal carcinoma | 2 | IIb | T2N1M0 | 45% | - |
| B4 | G | F | 45 | Breast | Invasive ductal carcinoma | 2 | IIa | T2N0M0 | 25% | + |
| C1 | H | F | 63 | Breast | Invasive ductal carcinoma | 3 | IIa | T2N0M0 | 90% | - |
| C2 | D | F | 58 | Breast | Invasive ductal carcinoma | 1 | IIa | T2N0M0 | - | +++ |
| C3 | I | F | 53 | Breast | Invasive ductal carcinoma | 1 | IIa | T2N0M0 | - | +++ |
| C4 | J | F | 34 | Breast | Invasive ductal carcinoma | 3 | IIb | T3N0M0 | - | ++ |

**Table S5. TMA core description from supplier.**

| 1. **Her2** | 1. **PR** |
| --- | --- |
| 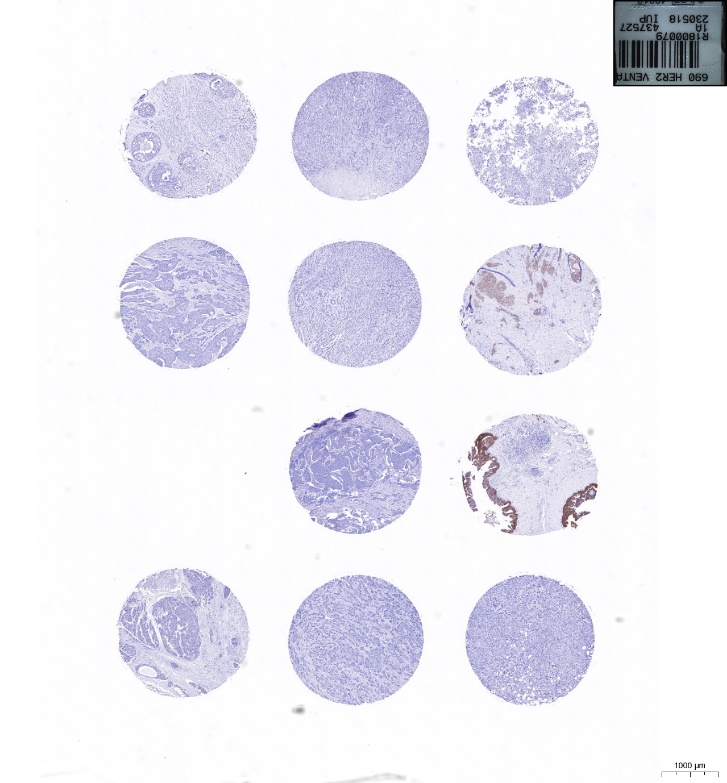 | 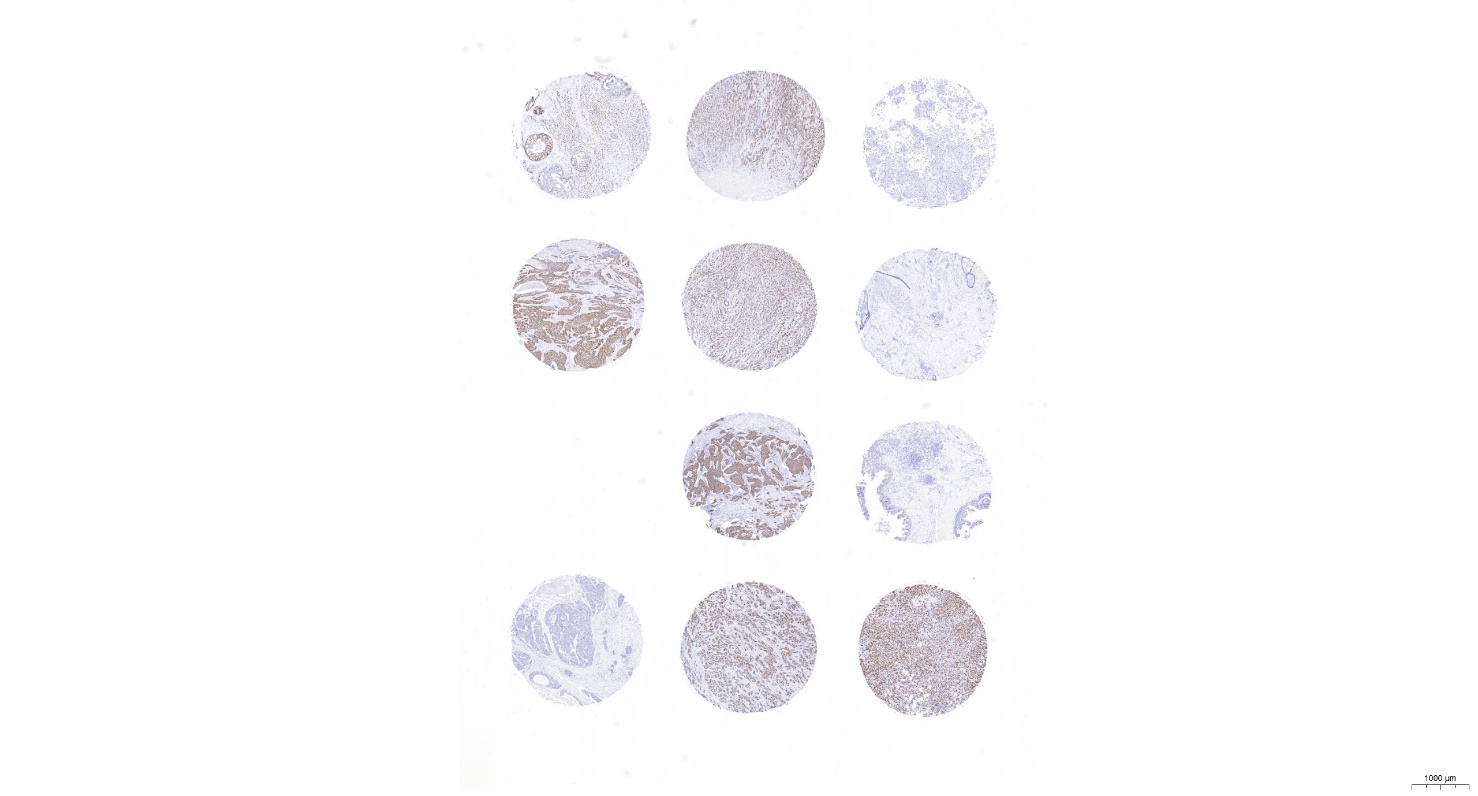 |
| 1. **ER** | 1. **Pan-CK** |
| 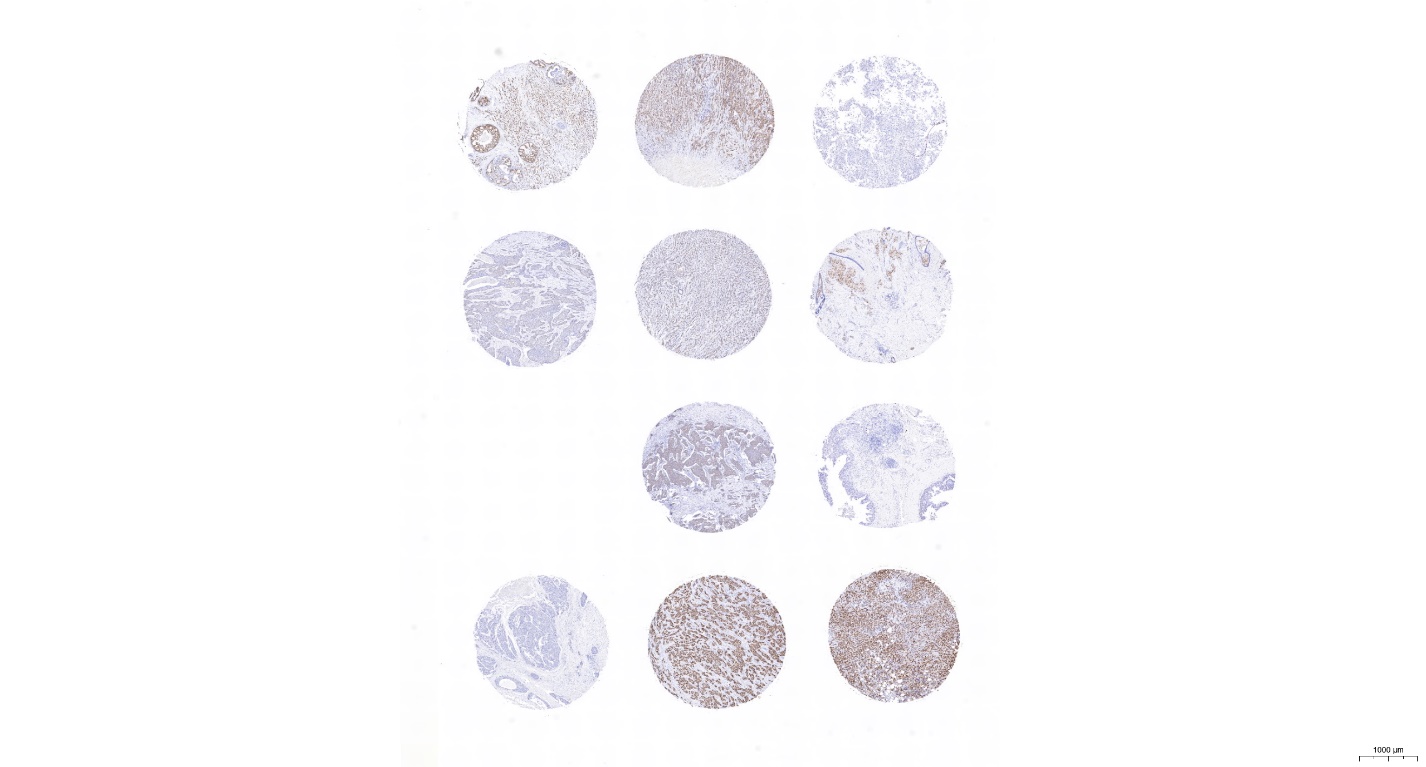 | 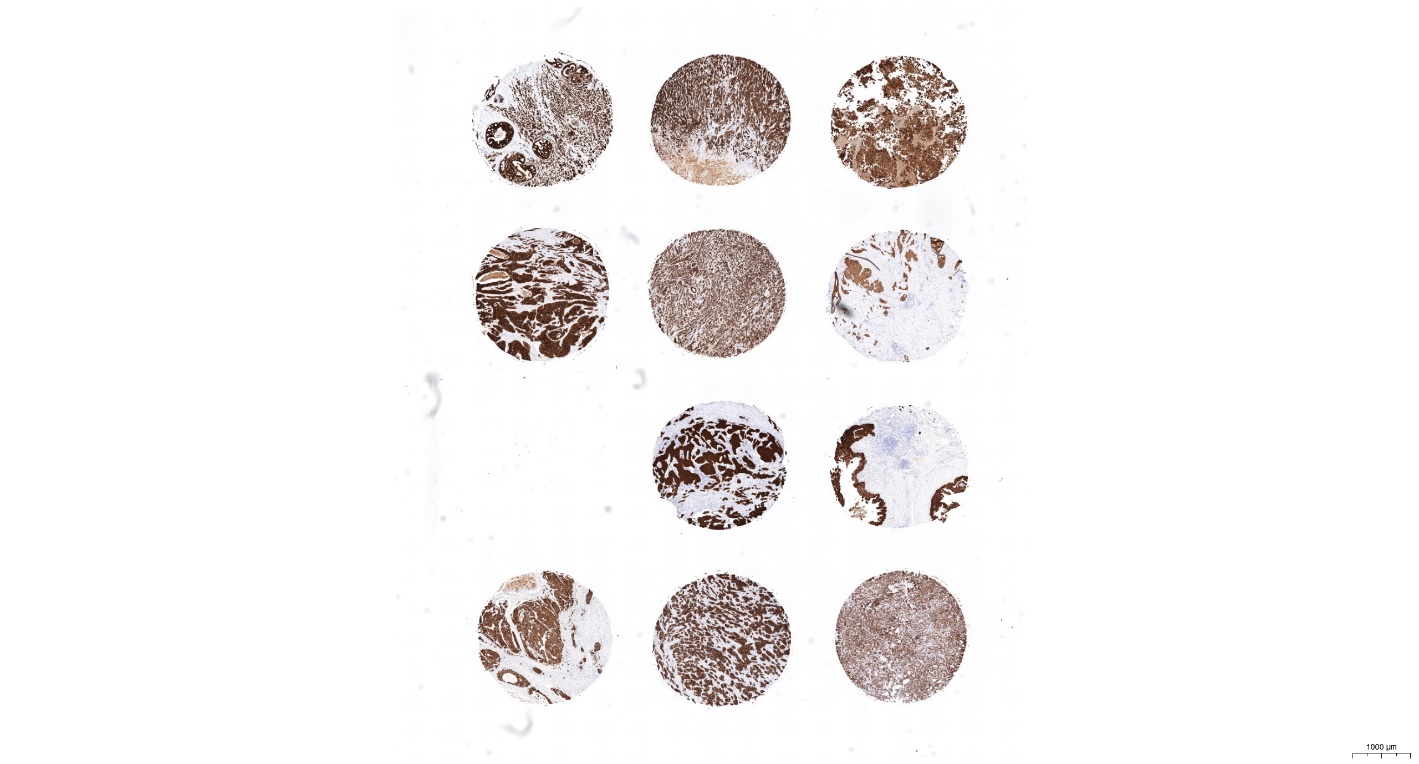 |

**Figure S6. IHC reference staining.** Brightfield image overview of chromogenic staining results performed on Ventana automat for (A) Her2, (B) PR, (C) ER and (D) pan-CK

**Figure S7 Reagent Delivery System (RDS).** Schematics of the experimental setup employed for the multiplexing experiments: (1) Pressurized Falcon tubes are used for common reagents, such as DIW and PBS; (2) Pressurized Eppendorf tubes are used to load specific reagents, such as antibodies, and TSA; (3) a valving system, operated via a computer, selects the reagents that are delivered into the chamber of reaction; (4) MTP/ tissue sample staining chamber.
